# Hippocampal regenerative medicine: neurogenic implications for addiction and mental disorders

**DOI:** 10.1038/s12276-021-00587-x

**Published:** 2021-03-30

**Authors:** Lee Peyton, Alfredo Oliveros, Doo-Sup Choi, Mi-Hyeon Jang

**Affiliations:** 1 Department of Molecular Pharmacology and Experimental Therapeutics, Mayo Clinic College of Medicine and Science, Rochester, MN USA; 2 Department of Neurologic Surgery, Mayo Clinic College of Medicine and Science, Rochester, MN USA; 3 Department of Psychiatry & Psychology, Mayo Clinic College of Medicine and Science, Rochester, MN USA; 4 Department of Biochemistry and Molecular Biology, Mayo Clinic College of Medicine and Science, Rochester, MN USA

**Keywords:** Adult neurogenesis, Addiction

## Abstract

Psychiatric illness is a prevalent and highly debilitating disorder, and more than 50% of the general population in both middle- and high-income countries experience at least one psychiatric disorder at some point in their lives. As we continue to learn how pervasive psychiatric episodes are in society, we must acknowledge that psychiatric disorders are not solely relegated to a small group of predisposed individuals but rather occur in significant portions of all societal groups. Several distinct brain regions have been implicated in neuropsychiatric disease. These brain regions include corticolimbic structures, which regulate executive function and decision making (e.g., the prefrontal cortex), as well as striatal subregions known to control motivated behavior under normal and stressful conditions. Importantly, the corticolimbic neural circuitry includes the hippocampus, a critical brain structure that sends projections to both the cortex and striatum to coordinate learning, memory, and mood. In this review, we will discuss past and recent discoveries of how neurobiological processes in the hippocampus and corticolimbic structures work in concert to control executive function, memory, and mood in the context of mental disorders.

## Mental health: Treatment hopes in the hippocampus

A region of the brain called the hippocampus and its connections to other parts of the brain via what are called cortico-limbic structures are implicated in a variety of mental health disorders. These disorders can be accompanied by reduced hippocampal volume. Mi-Hyeon Jang, Doo-Sup Choi and colleagues at the Mayo Clinic College of Medicine and Science, Rochester, USA, review the role of hippocampal and cortico-limbic neurobiology in memory and mood in mental disorders. They focus particular attention on the role of neurogenesis, the production and growth of new nerve cells and connections. Disrupted neurogenesis in the adult hippocampus is implicated in conditions including addiction, depression, schizophrenia and related psychotic disorders. Learning more about neural regeneration in the hippocampus could yield insights into mental health conditions and open new avenues toward developing drug-based treatments.

## Introduction

**Fig. 1:**
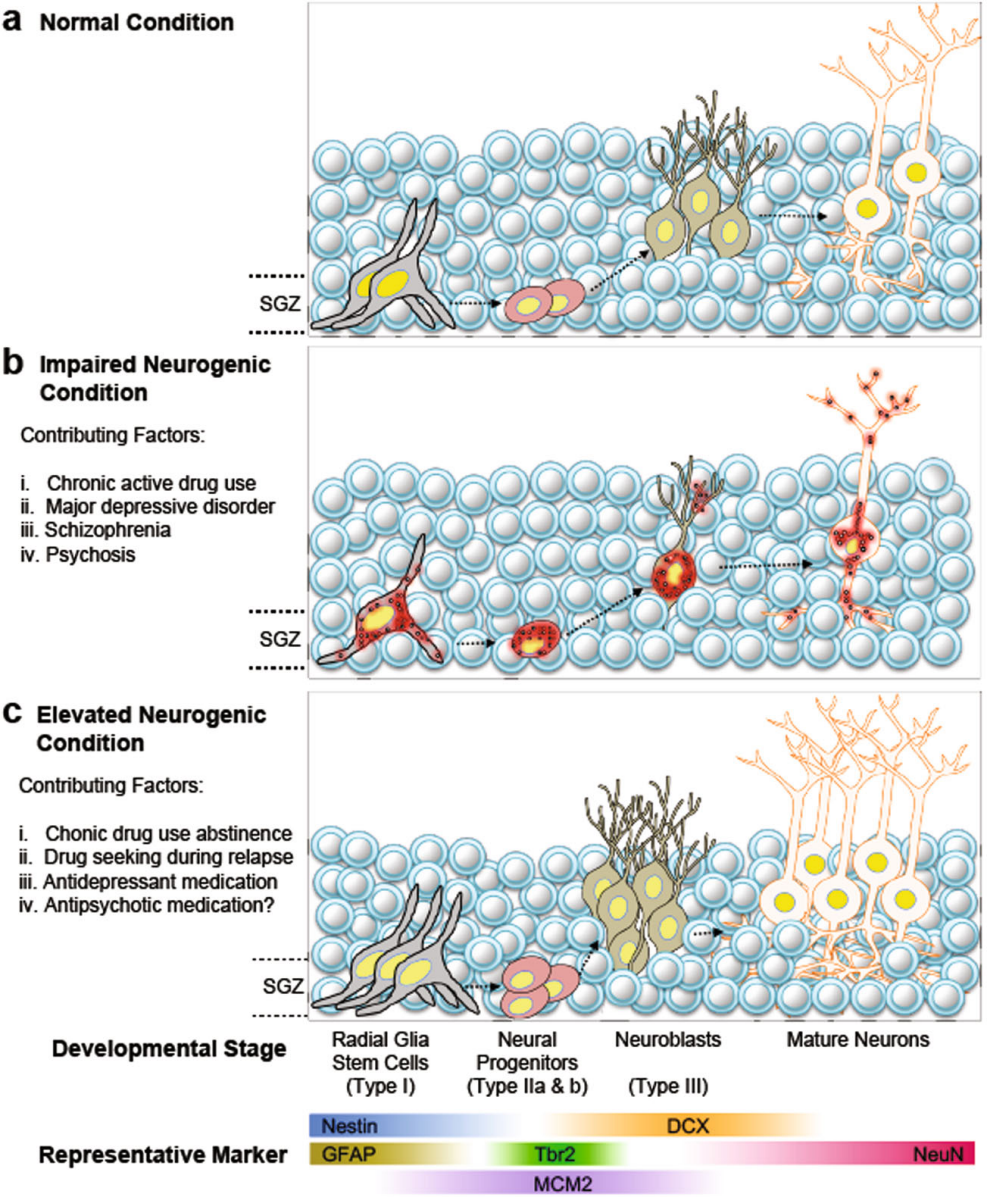
Adult neurogenic development is distinctly affected by neuropsychiatric disease. **a** Adult hippocampal neurogenesis is characterized by the development and maturation of stem cells into granular cell layer neurons in the dentate gyrus, which begin their functional journey as radial glia-like stem cells in the hippocampal subgranular zone (SGZ) and differentiate into multipotent progenitors (types I–III; see main text for details) and then into immature neurons/neuroblasts that eventually commit to a mature granular neuronal cell fate. These mature granular neurons form synapses with existing pyramidal neuronal circuitry to maintain cognitive function throughout life. **b** Conditions that impair the adult neurogenic process include: (i) actively heavy (binge) and/or chronic drug/alcohol use, (ii) major depression and related disorders, (iii) schizophrenia, and (iv) related psychoses. These aberrant neuropsychiatric conditions, all of which are characterized by significant psychosocial stress, likely induce apoptotic damage (i.e., red circles within cells, indicative of cellular breakdown) and subsequently limit the number of radial glia-like stem cells, multipotent progenitors, immature neurons, and mature granule cells in the hippocampal dentate gyrus. **c** Notably, several conditions increase or reverse the ability of biological processes to elevate the development and maturation state of adult neurons in the hippocampus. For example, while active intake of drugs of abuse is well known to decrease neurogenic development, paradoxically, (i) abstinence during recovery from drugs induces a rheostatic increase in neurogenic potential in the hippocampus. Surprisingly, during states of reward seeking, for example (ii) as a result of relapse in addiction, heightened adult neurogenesis is also observed, which may be maladaptive (e.g., continued drug seeking vs. seeking alternative behaviors to drug taking). Interestingly, other contributors to the increase in neurogenic potential in the context of neuropsychiatric conditions are (iii) antidepressant medications and potentially, (iv) antipsychotic medications.

Psychiatric illness is a prevalent and highly debilitating disorder that affects a significant percentage of the population in developed high-income countries^1^. While there is still much to learn regarding the neural circuitry and molecular mechanisms that are affected by neuropsychiatric disease, dysregulation of the corticolimbic circuit, which includes the cortex, striatum, and hippocampus^2^, has been implicated in mental disturbances that negatively affect learning, memory, and mood^3^. The hippocampus has long been implicated in psychiatric disorders due to its enduring plasticity throughout life and sensitivity to environmental changes^4^. Reductions in hippocampal volume have been reported in patients suffering from a variety of psychiatric disorders, including depression, addiction, and schizophrenia. Moreover, lesions of the ventral hippocampus in preclinical models recapitulate the characteristics of schizophrenia and other mental disturbances^4,5^. The hippocampus is a brain region capable of continuously generating newborn progenitor cells throughout adulthood, thereby giving rise to new neurons (Fig. 1a). The main prevailing neurogenic zones of the adult brain are the subventricular zone (SVZ) and the hippocampal subgranular zone (SGZ) of the hippocampal dentate gyrus (DG). The SVZ lines the lateral ventricles of the forebrain and contains progenitor cells poised for maturation into neurons within the olfactory bulbs^6^. In the hippocampus, the SGZ of the DG contains progenitor cells that mature into excitatory dentate granular cell neurons, which receive excitatory input from the entorhinal cortex^6,7^.

The discovery of adult hippocampal neurogenesis within the mammalian brain has long been controversial since Altman and Das’s^8^ original study, generally due to difficulties in the acceptance of the fact that progenitor cells give rise to new neurons in specific regions termed “neurogenic niches”^9^ (i.e., the SGZ and SVZ), and the relatively sparse evidence for neurogenesis in adult humans obtained in subsequent years. Over time, however, the concept of adult hippocampal neurogenesis has garnered intense interest. By the late 1990s, this process was characterized in Eriksson et al.’s seminal report, in which the authors described how new neurons in the DG of the human hippocampus are functionally relevant to spatial memory, episodic memory, and other cognitive functions^10,11^. Although numerous clinical and preclinical animal investigations have since documented the existence of adult hippocampal neurogenesis^6,12–15^, a recent study suggested that hippocampal neurogenesis may be extremely rare in the adult human brain after young adolescence^16^. Such discrepancies and contradictory findings on the existence of human hippocampal neurogenesis can likely be attributed to technical issues related to postmortem tissue processing. For example, Sorrells et al.^16^ used doublecortin (DCX) and Polysialylated-neural cell adhesion molecule (PSA-NCAM) as marker proteins to detect neurogenesis but failed to detect DCX/PSA-NCAM^+^ cells in the DG of individuals between 18 and 77 years of age and patients with chronic epilepsy, concluding that neurogenesis does not occur in the adult human hippocampus. In contrast, Boldrini et al.^12^, Moreno-Jiménez et al.^14^, and Tobin et al.^15^, utilized the same markers but clearly demonstrated the existence of neurogenesis. The most critical step in accurately detecting marker proteins is a tightly controlled postmortem delay (which is the time between the death of a person and fixation of the brain; <26 h), given that DCX can rapidly breakdown after death. Other critical variables that may affect adult hippocampal neurogenic expression are the timing of fixation and the available tissue processing technology (Moreno-Jiménez et al.)^14^, which may explain the relative absence of this marker in the tissues analyzed by Sorrells et al.

Although numerous studies have reported correlations between adult hippocampal neurogenesis and numerous pathophysiological conditions, including epilepsy, stroke, and neurodegenerative disorders, whether altered hippocampal neurogenesis contributes to psychopathology remains unclear and needs further study. An enduring question that underlies the study of adult neurogenesis in psychopathology is whether dysfunctional neurogenic changes occur as part of disease pathology or, alternatively, as an adaptive response that compensates for disease states generated by environmental stressors. Diminished cell proliferation within the DG and reduced hippocampal volume have been documented in the context of several psychiatric disorders (e.g., anxiety, depression, addiction, and schizophrenia)^17–19^. Interestingly, subpopulations of patients with major depression disorder (MDD) show decreased hippocampal volume and cognitive deficits^20^. Along these lines, studies utilizing volumetric magnetic resonance imaging (MRI) have consistently shown lower hippocampal volume and reduced gyrification in patients with schizophrenia^21–23^. While deficits in neurogenic capacity resulting from depression and neurodegeneration have been reported for some time, more recent discoveries have also implicated drug addiction in these deficits. For example, chronic use of cocaine^24^ and alcohol^25^ suppresses neurogenesis in rodents. Supporting these preclinical findings, multiple neuroimaging studies have found reduced gray matter volumes in the prefrontal cortices of cocaine-dependent patients compared with those of healthy controls^26^.

These studies bring to light the possibility that dysregulation of the adult neurogenic process may itself play a seminal role in the symptomatology of psychiatric disorders. Therefore, experimental characterization of the neurogenic process can engender the discovery of novel therapeutic targets in psychiatric disorders. Additionally, intensely researching the interaction between neurogenesis and psychiatric disorders can provide critical insight into the effector systems involved, which may increase the likelihood of developing successful treatments for psychiatric disorders. Consequently, we posit that the generation of new neurons throughout life is imperative to overall mental health, which is supported from the fact that (1) behavioral abnormalities mimicking the symptoms of diseases inhibit neurogenesis; (2) certain pharmacological treatments or behavioral interventions alleviate mental illness and enhance neurogenesis; (3) certain circumstances or environmental contexts increase the probability of developing mental illness, thus inhibiting neurogenesis; and (4) neurogenic potential is changed in patients already diagnosed with a psychiatric illness^27^. As such, we will review the role of adult neurogenesis within the context of alcohol addiction, depression, and schizophrenia, paying special attention to the molecular and cellular targets relevant to psychiatric disease and adult neurogenesis.

## Adult neurogenesis: a simplified overview

The process of adult neurogenesis is divided into four main stages: (1) proliferation of neural stem cells (NSCs) and neural progenitor cells (NPCs), (2) NSC/NPC migration from a specific neurogenic niche to areas of integration, (3) differentiation of NPCs into mature neurons, and (4) integration of cells into synaptic networks that can affect behavior and cognition^28^. NSCs and NPCs are multipotent, highly proliferative cells. In rodents, NSCs in the DG primarily reside in the SGZ, where four subtypes (type I, type IIa, type IIb, and type III) differing in morphology, proliferation rate, and protein expression profiles have been characterized. Type I NPCs resemble glia and have radial processes. Additionally, these type I NPCs express the intermediate filament protein nestin and glial fibrillary acidic protein (GFAP) and have a lower proliferation rate than other NPC subtypes^28^. In contrast, type IIa cells do not express GFAP, are not radial, and exhibit a much higher proliferation rate than type I NPCs. Type IIa cells express Tbr2 (ref. ^29^) and nestin and do not exhibit a radial glia-like morphology. Type IIb cells are unique in that they maintain expression of Tbr2 while showing commitment to a neuronal lineage, as indicated by the expression of the microtubule-associated protein DCX (Fig. 1a)^29^. Type IIb cells give rise to type III cells, which differ from the former in several aspects; the latter exhibit a round nuclear morphology but no longer express the stem cell marker nestin. Type III cells express DCX and PSA-NCAM and are fully committed to the postmitotic neuronal lineage, eventually expressing the mature neuron markers NeuN and calretinin^30^.

Neurogenesis in the SVZ within the lateral ventricles is similar to that in the DG, and only three cell types are implicated in the process (type B, type C, and type A). The cell types are analogous to those in the DG, with type B cells resembling type I cells in the SGZ with regard to morphology, protein expression profiles, and proliferation rate^31^. Type C cells are similar to type II cells in the SGZ, exhibiting no GFAP expression and a high proliferation rate. Interestingly, both cell types (A and B) are nestin^+^ and Sox2^+^, although type A cells (analogous to type III cells in the SGZ) are neuroblasts with the capability of migrating along the rostral migratory stream to the olfactory bulb^31^. Although they originate from different locations, NSCs in both the DG and lateral ventricles can produce cells with the capacity to differentiate into neurons and astrocytes. Generally, neuroblasts from the SVZ preferentially differentiate into olfactory bulb interneurons, while NSCs of the DG migrate to the granular cell layer, allowing for the integration of granular cells into the hippocampal circuitry through the formation of glutamatergic synapses with other granular neurons, interneurons, and pyramidal cells.

### Adult hippocampal neurogenesis: preclinical and clinical studies of addiction

Historically, the hippocampus has been accepted as a critical constituent of the limbic system, playing roles primarily in learning and memory. However, more attention is being paid to the hippocampus due to its involvement in the behavioral adaptability of mood, the associations between drug context, and the rewarding properties of drug abuse^32^. Additionally, the hippocampus is involved in the maintenance and acquisition of drug-taking behavior due to its ability to mediate associative memories of drug-taking, thereby regulating relapse upon re-exposure to drug-related contexts^19^. The anatomical location of the hippocampus allows this brain region to influence the brain reward pathways (dopaminergic reward circuitry) through afferent projections from the ventral striatum and ventral tegmental area (VTA). Further evidence of hippocampal involvement in the reward circuit was obtained from functional studies indicating that the hippocampal CA1, CA3, and DG subfields are manipulated after the firing of dopaminergic cells in the VTA and the discovery of a dedicated population of cells encoding reward in the hippocampus^33,34^.

Interestingly, recent studies have highlighted the importance of hippocampal neurogenesis in the regulation of reward seeking during abstinence for natural rewards. These seminal studies conceptually suggest that during periods of unpredictable reinforcement delivery (e.g., abstinence from natural reward/drug intake and prior to relapse when drug seeking is at its most intense), disruptions in the expectation of receipt of a natural sucrose reward foments impulsive response seeking behavior, which is associated with increased, albeit aberrant, neurogenesis^6,35^. This concept was confirmed by separate studies showing that abstinence from methamphetamine-related reward seeking increased aberrant hippocampal neurogenesis^36^. However, it is possible that addiction-related dysfunctional neurogenesis may be drug-dependent, as there is controversy regarding whether opiate seeking vs. opiate taking influences neurogenesis during abstinence from drugs of abuse^37,38^ (Fig. 1c).

Additional research on how readily available substances, such as alcohol and, to a lesser extent, cannabis, are able to affect neurogenic potential is crucial. Furthermore, given that both alcohol and cannabis are legal or soon-to-be-legal substances that can be used recreationally, it is imperative to understand the neurobiological mechanisms of action to identify novel targets and treatment strategies for addiction to these compounds.

### Alcohol

Ethyl alcohol, due to its ease of acquisition and cultural importance, is one of the most extensively studied substances with regard to addiction. Excessive alcohol consumption is a hallmark characteristic of alcohol use disorder (AUD). Studies on adult neurogenesis and alcohol consumption have generally indicated a negative correlation between the two, with reduced neurogenesis resulting from chronic alcohol abuse. However, as with other drugs of abuse, the effect of alcohol on adult neurogenic potential varies with dosage, duration of exposure, and intake pattern. Translational studies investigating neural adaptation associated with AUD have been integral to elucidating the effects of the disease. Due to greater advancements in technology used to assess brain function in vivo, a more consistent understanding of the effects of alcohol on the hippocampus has emerged. For instance, fMRI of adolescent AUD patients has shown a reduction in hippocampal volume, with early-onset alcohol consumption potentiating these deficits^39^. Perturbations in NSC populations by alcohol may be associated with decrements in hippocampal volume in AUD patients since the production and turnover of these cells ultimately govern the total number of neurons and the subsequent volume of this brain region^10^. Despite this association, improved imaging techniques, perhaps in combination with cell-type specific optogenetic manipulation, will be helpful to identify the specific subregions of the hippocampus or specific cellular populations that are uniquely susceptible to alcohol abuse. Notably, recent clinical reports have shown that transmagnetic deep brain stimulation has promise in reducing nicotine consumption and cravings^40,41^. Therefore, it may soon be feasible to use a minimally invasive clinical optogenetic approach (perhaps in combination with deep brain stimulatory currents) to identify or manipulate the functional neurogenicity of specific hippocampal cell populations susceptible to alcohol damage in the future^42^.

The investigation of postmortem human brains in conjunction with studies of preclinical models of addiction has provided key insights into the effects of alcohol on DG neurogenesis, as dose, duration, and frequency of alcohol exposure can differentially affect hippocampal neurobiology. Animal model studies of moderate chronic alcohol exposure have indicated that ethanol is toxic to hippocampal granule cell neurons but has little effect on NPC proliferation^43^. Paradoxically, long-term voluntary alcohol self-administration in the two-bottle choice test enhances NPC proliferation but had no effects on the differentiation or survival of these cells in mice^44^. Interestingly, numerous studies have suggested that alcohol may kill NSCs or NPCs in long-term alcohol exposure models. These studies suggest that neuronal cell death induced via alcohol is dose- and duration-dependent, as sustained blood alcohol concentrations (BACs) >200 mg/dl (0.20 g %) are known to induce neurodegeneration^39,45^. In line with these reports, hippocampi of controls with no history of alcohol consumption compared to hippocampi isolated from deceased donors with a known history of prolonged alcohol abuse showed decreases in granular cell number and granular cell layer volume^46^. Subsequent immunostaining for markers of cell proliferation (Ki67), stem/progenitor cells (Sox2), and immature neurons (DCX) revealed significant deficits in the expression of all markers in the DG in donors with a prolonged history of alcoholism compared with healthy controls^47^ (Fig. 1b).

Several possible mechanisms underlying the effect of alcohol in disrupting the neurogenic process have been proposed. The cyclic adenosine monophosphate (cAMP)–responsive element binding (CREB) protein signaling pathway is known to be sensitive to alcohol^45^. Indeed, this pathway is a main contributor to neurogenesis through its regulatory effects on new neuron proliferation, differentiation, and survival^39^. Two main targets of the cAMP–CREB pathway are neuropeptide Y (NPY) and brain-derived neurotrophic factor (BDNF), both of which are important promotors of adult hippocampal neurogenesis^48,49^. In the DG, NPY is expressed in a subpopulation of GABAergic interneurons, and its receptor, Y1, is expressed in SGZ NPCs. BDNF promotes the survival and differentiation of NPCs, and some of the highest levels of BDNF are found in the hippocampus and DG. Multiple lines of evidence have shown that alcohol exposure has detrimental effects on hippocampal BDNF expression, reducing neurogenesis and promoting depressive-like behaviors^50,51^. Given the direct role of cAMP–CREB signaling in regulating adult hippocampal neurogenesis^45^, it is conceivable that alcohol adversely alters neurogenesis through CREB signaling.

### Cannabis

Cannabis use is increasing widely as legalization of its recreational use increments worldwide. As approximately 2.5% of the world population consumes cannabis, it is important to consider the effects of prolonged use on the neurobiology of cognition and homeostatic brain function. Cannabinoids include the active components of the plant *Cannabis sativa*, endocannabinoids, and synthetic cannabinoids. The neural cannabinoid system constitutes a complex series of receptor–ligand and receptor–receptor interactions that include various signaling pathways, such as growth factor receptor and G-protein receptor signaling, which control a wide variety of physiological processes, including adult neurogenesis^28^. Cannabinoids exert their effects via activation of G protein-coupled cannabinoid receptors type 1 and type 2 (CB_1_ and CB_2_, respectively), which are localized throughout the central nervous system (CNS), including on astrocytes, microglia, and neurons. Studies have suggested that various cannabinoid ligands control cell genesis and overall neurogenesis in the mammalian brain^28,52^. Therefore, NPCs expressly regulate endocannabinoid systems to produce endogenous cannabinoids^53^, highlighting the potential of the cannabinoid system to exert regulatory control over adult neurogenesis.

A recent study in which stressed young adult mice voluntarily inhaled cannabis smoke for 2 months and hippocampal neurogenesis in the DG was assessed, demonstrated that smoking cannabis regulates adult hippocampal neurogenesis^52^. Interestingly, the results of this study suggested that NPC proliferation in mice was unaffected by cannabis smoke, whereas the number of DCX^+^ cells in the DG was decreased, dendritic morphology was altered, and migration was perturbed in cannabis-exposed mice compared to untreated controls^52^. This finding is comparable to others indicating that the endocannabinoid system plays an important role in regulating cell proliferation, migration, and differentiation through differential activation of CB_1_/CB_2_ receptors^54^.

## Neurogenesis and major depressive disorder

Depression is a prevalent neuropsychiatric disorder afflicting approximately 17–20% of the world population, resulting in increased mortality, social/economic burden, and personal suffering^55^. The neurogenesis hypothesis suggests that adult neurogenesis within the SGZ of the DG is negatively regulated by stressful stimuli and positively regulated by antidepressant drugs^56^. Additionally, it posits that perturbations in the rate of neurogenesis are critical in the pathology and treatment of depression, which is suspected to be multifaceted. The reduction in hippocampal volume (particularly gray matter volume) in patients diagnosed with depression highlights the potential for alteration of adult neurogenesis in the development of the disease^20^. A meta-analysis of 32 publications indicated that hippocampal volume is significantly reduced in individuals who experience a major depressive episode lasting more than 1 year or >2 years of overall illness in their lifetimes, suggesting that volume reductions are attributes of the disease rather than contributing risk factors^57^. While it is argued that many factors other than a decrease in the number of neurons may account for a decrease in hippocampal volume, the prevailing evidence suggests a role for neurogenesis in the etiology of depression (Fig. 1b).

Dual diagnosis (diagnosis with both a mental disorder and substance-use disorder) is a common diagnosis for people with MDD. Bidirectional cooccurrence is common for MDD and AUD^58^. This co-occurrence is observed in other industrialized nations, as the incidences of MDD and AUD co-occurrence in the population in France and United States are 14% and 9.5%, respectively, and MDD+AUD patients displaying a more severe depression profile than patients experiencing MDD alone^58^. The conclusions of this retrospective study are not surprising, as it is common for patients with depression to self-medicate^59^. Given that alcohol consumption dampens the neurogenic process, chronic consumption of alcohol presumably worsens the deficits in neurogenesis that accompany depression.

Despite the controversy related to the role of neurogenesis in depression, numerous preclinical studies support this concept, showing that neurogenic stimuli can be modulated via antidepressant medication and wheel running physical exercise^60^ (Fig. 1c). Interestingly, a positive correlation has been shown between promotion of neurogenesis by the selective serotonin reuptake inhibitor (SSRI) fluoxetine and exercise, with rodents displaying reduced signs of depression/anxiety after pharmacotherapy or running exercise^61^. Direct supporting evidence was first provided by Mateus-Pinheiro et al., who demonstrated that abolishment of adult neurogenesis gives rise to depressive-like behavior, including decreased consumption and preference for a natural reward, as well as decreased mobility time in the forced swim test, which assessed depressive-like behavior in rodents. Importantly, these behavioral deficits were rescued by fluoxetine administration^62^. These investigations have been corroborated by subsequent studies showing that selective abolishment of adult hippocampal neurogenesis alters the antidepressant efficacy of fluoxetine administration, indicating that adult neurogenesis is required for the antidepressant effect of fluoxetine^63^. In contrast, increasing adult hippocampal neurogenesis using selective conditional deletion of the pro-apoptotic bax gene in adult-born neurons is sufficient to reduce anxiety and depression-like behaviors in a mouse model of stress^64^. Notably, chronic social defeat stress (CSDS) potentiates depressive-like behavior and neurogenic impairments, both of which are rescued by tetracyclic triterpenes (found in sapogenins). Tetracyclic triterpene administration activates BDNF signaling and neurogenesis in the hippocampus^55^. Mechanistically, neurogenesis was recently shown to confer resilience to chronic stress by decreasing the neural activity of mature granule cells located in the ventral hippocampal DG, a brain region implicated in mood regulation^65^. This study also showed that chemogenetic inhibition or activation of adult neurogenesis in the ventral hippocampus DG causes susceptibility or resilience, respectively, to social defeat stress. Moreover, in vivo calcium imaging has revealed that neurogenesis leads to decreased activity of stress-responsive granule cells that are preferentially activated during stressful circumstances and that both of these effects within the DG are necessary for stress resilience^65^. In summary, this study emphasizes the importance of adult hippocampal neurogenesis in the etiology of depression.

Recently, the role of the Wnt (wingless-related integration site) signaling pathway in mood disorders has garnered more attention, as unabated Wnt signaling is imperative for hippocampal neurogenesis^66^. A full summary describing in detail the contributions of Wnt signaling to adult hippocampal neurogenesis is not within the purview of this review and is discussed elsewhere^66^. However, it is sufficient to say that manipulation of members of the canonical Wnt pathway (β-catenin, glycogen synthase kinase-3β, Wnt3, secreted frizzled related protein 3, and Dickkopf 1) has been shown to impact the target trajectory of newborn neurons within the DG^66^. Indeed, a genome-wide association study (GWAS) examining differentially expressed genes between patients diagnosed with MDD and healthy controls identified a key single-nucleotide polymorphism (SNP) (rs1969253) in *DVL3*, a component of the Wnt signaling pathway^67^. Accordingly, SNPs in *DVL3* display the strongest association with MDD and are increased in patients currently experiencing MDD^67^.

## Neurogenesis and psychosis

Schizophrenia and related psychotic disorders are extremely debilitating neuropsychiatric conditions characterized by a loss of contact with reality, and the median incidence of these disorders in the general population in the United States is 3–5 per 1000 (ref. ^68^). These results are supported by more recent studies showing that the incidence of psychosis is related to sex and immigration status and that a higher incidence of psychosis is associated with lower socioeconomic level as well as exposure to traumatic experiences during childhood and adolescence^69,70^. Although psychosis is the most prominent “positive” symptom (e.g., hallucinations and delusions) of schizophrenia, it is one of the three main symptoms required for diagnosis, as “negative” emotional symptomatology (e.g., reduced motivation, emotional expression, and poor planning ability) and cognitive dysfunction (e.g., deficits in attention, concentration, and memory) are also diagnostic criteria for schizophrenia. This debilitating disease often strikes young individuals on the cusp of a promising future during a period where the developing brain encounters stressful social contexts (i.e., university education, military service, entry into the workforce, traumatic experiences, etc.), which in combination with a genetic predisposition, may tip the balance toward emergence of a first psychotic episode^71^. Given that intracranial brain volume stabilizes and is maintained in early- to mid-adolescence^72^, it is not illogical to assert that schizophrenia is a neurodevelopmental disorder. In support of this hypothesis, several reports have demonstrated distinct deficits in hippocampal volume in young patients in mid-to-late adolescence following a first psychotic episode^73,74^. Unfortunately, during this critical juncture of adolescent neurodevelopment, when intracranial brain substructures are still undergoing maturation and development, hippocampal volume does not seem to be restored by antipsychotic drug treatment^74^. While the average age of onset of the initial psychosis episode occurs after early adolescence in females (early twenties to early thirties) and males (late teens to late twenties), clinical epidemiological investigations have revealed that psychosis manifestations are often preceded by poor academic performance^75,76^.

If schizophrenia patients have cognitive deficits along with psychotic symptoms, could impaired neurogenesis be a contributing factor to psychosis-associated cognitive dysfunction? Indeed, the postmortem brains of patients with schizophrenia exhibit distinct reductions in Ki67^+^ cells and PSA-NCAM expression in the hippocampal DG, which suggests that adult neurogenesis is impaired^17^. Other studies have identified hippocampal volume reductions in schizophrenia patients who harbor a familial SNP in the allele coding for the G-protein coupled receptor SREB2, and notably, SREB2 transgenic overexpression in animal models recapitulates schizophrenia-like behavioral and neurogenic deficits that can be reversed by deletion of SREB2 (ref. ^77^). Interestingly, observations of intracranial and hippocampal volume deficits in the postmortem brains of schizophrenia patients have given rise to the hypothesis that stressful life events and subsequent psychosis may in fact result in cell death^74^. Supporting this notion, a recent report examined the effects of poly (ADP-ribose) polymerase-1 (PARP-1) deletion in cultured NSCs and found impaired neuronal lineage cell differentiation with concomitant PI3K-Akt-ERK-FOXO1-dependent increases in astroglial differentiation. Importantly, the authors demonstrated that a mouse model of constitutive PARP-1 deletion recapitulates schizophrenia-like impairments in NSC proliferation, memory, and prepulse inhibition (PPI)^78^. Therefore, the observed intracranial deficits exhibited by schizophrenia patients may be due to abnormal cell death resulting from the aberrant psychosis that is characteristic of the disease.

While much remains to be explored regarding adult neurogenic disruptions in patients with schizophrenia, animal models utilized in the study of this condition have revealed that disrupted in schizophrenia (DISC1) is critically involved in axonal targeting of mossy fiber projections and synaptic contact formation, as DISC1 knockdown disrupts these adult neurodevelopmental processes^79^. Notably, impaired axonal targeting and diminished synaptic contacts have also been observed in the postmortem hippocampi of schizophrenia patients^80^, suggesting that DISC1 is an important contributor to the etiology of schizophrenia. The atypical antipsychotic drug clozapine alleviates these aberrant alterations, and additional research on DISC1 has elucidated its potential to regulate neurogenic capacity and schizophrenia-like impairments in hippocampal-related cognitive tests^81,82^. Mechanistically, selective inducible expression of DISC1 in astrocytes was shown to result in decreases in the hippocampal expression of the NPC marker MCM2 and dendritic outgrowth of newborn neurons, both of which are restored by d-serine administration^83^. These findings suggest that astrocytes are instrumental in the metabolic regulation of neurogenic function and functional cognition, which are notoriously impaired in schizophrenia^83^. Surprisingly, the potential involvement of astrocytes in regulating neurobiological processes that are disrupted in schizophrenia highlights the involvement of glutamate neurotransmission in the etiology of schizophrenia and related psychotic disorders^81,84^. Recently, Provenzano and colleagues observed high hippocampal glutamate levels and a large degree of hippocampal atrophy in patients at clinically high risk of developing schizophrenia^73^.

Glutamate is an excitatory neurotransmitter that binds the *N*-methyl-d-aspartate receptor (NMDAR) and plays an essential role in synaptic plasticity, neurogenesis, and adequate neuronal function via promoting metabolic homeostasis in the brain. Therefore, evidence for glutamate’s involvement in the etiology of schizophrenia suggests that dysfunctional glutamate neurotransmission can be a contributor to psychosis development. Consequently, kynurenic acid (KYNA), an endogenous NMDAR antagonist and metabolic product of tryptophan–kynurenine metabolism, was recently detected in the cerebrospinal fluid (CSF) of Alzheimer’s disease patients, linking hippocampal neurogenic disturbances to aberrant kynurenine metabolism^85^. In line with the neuroinflammatory hypothesis of schizophrenia, observation of inflammation-mediated increases in the expression of IL-1β, an inflammatory cytokine effector released by microglia, the brain’s immune surveyor and defender^86^, have been demonstrated to decrease the levels of neural lineage differentiation markers DCX and MAP1 in human cortical and hippocampal cultures while promoting the proliferation of astroglial cell types^87,88^. Moreover, following LPS-induced neuroinflammation, in situ hybridization of mRNA specific for the enzyme kynurenine aminotransferase II (KATII), which encodes KYNA, has revealed increased KATII expression in the rostral migratory stream, SVZ and hippocampal neurogenic niche^89^. It has been well established that neuroinflammation impairs cognitive function in animal models^90,91^. Therefore, it is hypothesized that neuroinflammatory mediators, which are uniquely involved in the neurodevelopment of schizophrenia^92^, seemingly include components of kynurenine metabolism^86^. Not surprisingly, the anti-inflammatory drug minocycline, which coincidentally is also an NMDAR antagonist, has shown promise in reversing excessive synaptic pruning and engulfment by microglia in schizophrenia patient-derived neuronal and microglial cocultures^93^. Importantly, microglia and their effectors (e.g., chemokine CCL-11) are pivotal cellular mediators of neuroinflammation and can contribute to adult hippocampal neurogenesis as well as homeostatic synaptic pruning. Therefore, microglia are important cells in the clinical development of schizophrenia and psychosis^94,95^.

The involvement of inflammation in schizophrenia and related psychoses has attracted increasing interest. Maternal immune activation animal models of schizophrenia can be generated by administering LPS or polyinosinic:polycytidylic acid (Poly I:C), which triggers a maternal immune response to bacterial or viral pathogens, respectively. Subsequent prenatal and postnatal cytokine-induced inflammatory mediators (TNFα, IL-8, IL-6, IL-1β, etc.) are speculated to play a role in the development of schizophrenia-like behaviors in adult offspring^81^, including disruptions in neurogenic function, sensorimotor gating, and hippocampal-dependent attention and working memory^96^. Moreover, antipsychotic medications have been reported to have limited ability to reverse the aforementioned behavioral impairments observed in maternal immune activation animal models^84^. However, regarding the restorative effects of antipsychotic medications on neurogenic function, further research is warranted, as the neurogenic benefits of typical (e.g., haloperidol) vs. atypical (e.g., clozapine) antipsychotic medications are inconclusive.

In summary, increasing evidence demonstrates that schizophrenia and psychosis-related conditions have a strong neurodevelopmental etiology, with adult neurogenic development and function being distinctly and aberrantly affected (Fig. 1b). Consequently, disruption of adult neurogenic development negatively affects cognitive function and possibly exacerbates psychosis, as the two are seemingly intertwined with regard to symptom manifestation. Continued research on adult neurogenesis, excitatory neurotransmission, and metabolic bioenergetics in combination with immune-mediated mechanisms will pave the way for a better understanding of the etiology of schizophrenia. Therefore, a mechanistic understanding of psychosis will enhance the discovery of therapeutic molecular targets and improve the quality of life of people suffering from this debilitating disease.

## Emerging pharmacological strategies and tools for the study of neurogenesis

Remarkable strides have been made in research on the application of NSCs and NPCs for the development of potential stem cell-based therapies for neuropsychiatric disorders, CNS injury, and neurodegenerative diseases. As such, regenerative medicine provides a new opportunity for psychiatric disorder treatment. For instance, in the CNS, under diseased or injured conditions, transplanted NPCs tend to differentiate into astrocytes. Application of small molecules to facilitate the differentiation of these progenitor cells toward the desired cell fate will be a feasible therapeutic option in the near future.

Some of these novel small molecules are members of the bromodomain and extraterminal (BET) family of proteins, which consists of Brd2, Brd3, Brd4, and testis-specific BrdT, which are epigenetic readers of the acylated histone code of chromatin^97^. Interestingly, BET proteins have been demonstrated to regulate the gene expression of multiple genes implicated in tumor cell growth, inflammatory responses, and cardiac hypertrophy^98^. Since BET proteins are strongly implicated in numerous disease states, selective^98^ small-molecule inhibitors of BET bromodomains can prevent the interaction between BET proteins and acylated histones. Accordingly, a small-molecule cocktail comprising BET-inhibiting compounds increased the direct conversion of fibroblasts into neurons^98^, suggesting that these compounds can efficiently reprogram other cell types into functional neurons, indicating that this cocktail may be a potential replacement therapy for neurological disorders. In addition, BET bromodomain inhibition was shown to promote neurogenesis while decreasing gliogenesis in NPCs^98^. Therefore, small molecules targeting BET family proteins may be useful for promoting endogenous neuronal regenerative capacity in the context of a broad range of mental disorders that are associated with impaired neurogenesis, such as depression and schizophrenia.

Undoubtedly, the emerging use of human brain organoids and pluripotent stem cells with the capability to self-organize and develop three-dimensional organ-like structures will likely prove to be useful in the discovery of therapeutics for neuropsychiatric disease^99^. Moreover, utilization of these novel research models will help elucidate the molecular underpinnings and mechanisms underlying the modulation of adult neurogenesis and its involvement in psychiatric disorders. Recent developments in organoid technologies and strategies have enabled remarkable, although limited, recapitulation of human neurodevelopment. The ontology of human corticolimbic structures and function is highly complex, proceeding through cycles of NPC and NSC proliferation, differentiation, migration, and apoptosis, all of which organoid models are able to recapitulate. In line with this, comparison of the development of 3D cerebral organoids derived from adult schizophrenia patients with that of organoids derived from healthy controls has already been utilized to identify aberrations in NPC proliferation^100^. Despite the extraordinary promise brain organoids offer in recapitulating human-specific developmental phenotypes, epitomized by the generation of outer SVZ-like progenitor cells, these models are not without drawbacks. Hypoxia leading to cell death (diffusion limit) results in outer SVZ shrinkage and disruption of radial glial scaffolds in prolonged culture, which prevents the migration required for cortical lamination^99^. To overcome such hurdles, new methodologies have been developed, including sliced neocortical organoids (SNOs), which overcome diffusion limits and possess better resolution of the more superficial and deeper cortical layers^101^, thus enabling organoids to be used in prolonged culture. Additionally, the SNO system has demonstrated utility in the study of the molecular mechanisms that control human cortical neuron subtype fate in psychiatric disorders^101^.

## Conclusion

Mental health disorders encompass a wide variety of ailments, which include addictive, depressive, and psychotic behaviors, all of which are extremely debilitating (Fig. 1). A broader understanding of the mechanisms regulating adult hippocampal neurogenesis in the context of neuropsychiatric disorders will allow for the exploration of new avenues in the design of therapeutics or potential personalized drug regimens aimed at curtailing symptoms. Furthermore, studies employing a combination of animal models, advanced brain imaging, optogenetics, brain stimulation, and cutting-edge human-derived brain organoids will be imperative for improving the understanding of the genetic or environmental factors that contribute to each disease state. Due to the complexity of the role of neurogenesis in mental disorders, it is necessary for investigators to employ interdisciplinary approaches combining genetics, highly validated animal and culture models, and pharmacology to understand these disorders and identify strategies to alleviate neuropsychiatric symptoms to improve the quality of life of patients suffering from these conditions.
